# Lymph Nodal Yield and Prediction of Mortality in Oral Squamous Cell Carcinoma Patients

**DOI:** 10.1002/ohn.70040

**Published:** 2025-10-17

**Authors:** Christian Mirian, Lasse Rehné Jensen, Mathias Hald, Davide Mattavelli, Cesare Piazza, Linda Feeley, Patrick Sheahan, Marcel Oehme, Christian Freudlsperger, Julius Moratin, Therese Ovesen

**Affiliations:** ^1^ Department of Otorhinolaryngology, Head and Neck Surgery Gødstrup Hospital Herning Denmark; ^2^ Department of Otorhinolaryngology Aarhus University Hospital Aarhus Denmark; ^3^ Unit of Otorhinolaryngology ‐ Head and Neck Surgery, Department of Medical and Surgical Specialties, Radiological Sciences and Public Health University of Brescia ASST Spedali Civili of Brescia Brescia Italy; ^4^ Department of Pathology Cork University Hospital Cork Ireland; ^5^ ENTO Research Unit, College of Medicine and Health University College Cork Cork Ireland; ^6^ Department of Otolaryngology ‐ Head and Neck Surgery, South Infirmary Victoria University Hospital Cork Ireland; ^7^ Department of Surgery University College Cork Cork Ireland; ^8^ Department of Oral and Cranio‐Maxillofacial Surgery Heidelberg University Hospital Heidelberg Germany; ^9^ Department of Clinical Medicine Aarhus University Aarhus Denmark

**Keywords:** head and neck, LNY, lymph nodal yield, oral squamous cell carcinoma, OSCC, survival

## Abstract

**Objective:**

Lymph nodal yield (LNY) is increasingly used in oral squamous cell carcinoma (OSCC) research as a prognostic factor and quality metric but remain to be validated across independent OSCC cohorts.

**Study design:**

Retrospective cohort study.

**Setting:**

Primary OSCC patients (n = 1080) who underwent a neck dissection, collected from three sites: Brescia, Cork, and Heidelberg.

**Methods:**

The accuracy of LNY in predicting 3‐ and 5‐year mortality was evaluated using the area under the ROC curve (AUC). Binomial regression models, adjusted for LNY and various confounders, were used to cross‐validate predictive performance across independent cohorts. A model estimating the population‐average treatment effect of LNY was used to estimate the effect of what *would have been* observed if the OSCC patients *had been* randomly assigned to have specific ranges of LNYs removed, irrespective of their individual characteristics. Mean LNY was estimated between the three cohorts.

**Results:**

ROC curve analysis demonstrated that LNY did not improve prognostic accuracy in predicting 3‐ and 5‐year mortality risks. Cross‐validation across independent cohorts showed that adding LNY to the binomial regression models did not improve predictive accuracy. Using the LNY range of 33 to 44 as a reference, the average treatment effect model found no significant difference in the 3‐year risk of death across LNY groups (LNY: 2‐17, 17‐25, 25‐33, and 44‐119) for pN‐positive patients.

**Conclusion:**

We found no improvement in the prognostic accuracy for mortality when considering LNY in different settings. Substantial inter‐center variability and lack of consistent survival benefit challenge the use of LNY as a prognostic or surgical quality metric.

Lymphadenectomy is key to controlling locoregional disease and improving overall survival outcomes. For head and neck cancer patients, the purpose of neck dissection is to remove lymph nodes containing either detected metastases or suspected for metastases, while balancing the associated risks of surgical toxicity.

Various patient‐, treatment‐, and center‐specific factors may impact the total number of lymph nodes yielded (LNY). In patients undergoing neck dissection, factors such as body mass index, prior radiotherapy, and the operating surgeon, are associated with variations in the total LNY.^1^ Variability in factors such as surgeon experience complicate drawing robust conclusions about the association between LNY and survival outcomes, as these inconsistencies inevitably leads to heterogeneity that is difficult to adequately account for, particularly across studies, but also within individual studies. In other surgical disciplines, higher age and more advanced disease stages have been described as factors that also influence total number of LNY.^2^

In the head and neck cancer literature, LNY is frequently reported as a predictor of survival outcomes and is widely used as a prognostic marker.^3^, ^4^, ^5^, ^6^, ^7^, ^8^, ^9^, ^10^, ^11^, ^12^, ^13^, ^14^ One common application is the use of LNY cut‐off values, with a minimum yield of 16 to 22 lymph nodes often proposed to predict favorable outcomes. In oral squamous cell carcinoma (OSCC) research, the 18 LNY cut‐off (18LNY) has gained widespread use. The 18LNY is being reported to differentiate OSCC risk groups and proposed as a minimum yield for “defining standard care” for selective neck dissection based on multivariable analysis of pooled data from independent centers.^15^, ^16^

Although a range of clinical and histopathological factors are frequently considered in adjusted regression analyses, measures of predictive accuracy and cross‐validation remain insufficiently applied to address the hypothesis that LNY improve prognostication.

Our objectives were to (1) assess factors associated with the total number of LNY across three European OSCC cohorts, to (2) evaluate the prognostic value of LNY and 18LNY within specific strata, and to (3) estimate the population‐average treatment effect of LNY across the independent cohorts using a counterfactual framework.

## Material and Methods

### Study Design

We conducted a retrospective cohort study using clinical data from 1080 patients with primary OSCC who underwent neck dissection. The study population was drawn from three independent European centers: Heidelberg (Germany), Cork (Ireland), and Brescia (Italy).

### Study Setting

The study included patients treated between 1999−2017 (Brescia), between 2011 and 2020 (Cork), and between 2011 and 2021 (Heidelberg). Data for the present study was collected in 2019 and analyzed in 2024. All included patients had pathologically verified primary OSCC and underwent elective or therapeutic neck dissection ‐ irrespective of extent or subtype of neck dissection (eg, selective, modified radical, or radical). However, detailed information on dissection type was not systematically available and is acknowledged as a limitation. Neck dissections performed as salvage therapy were not included in the study.

Data collection was performed retrospectively at each participating center by the co‐authors. No standardized data collection framework was used at the outset. The final data were received fully anonymized. Local Institutional Review Board approvals include: (1) NP‐2066‐Study WV‐H&N, approved by the Comitato Etico Provinciale della Provincia di Brescia; (2) approval from the Cork Clinical Research Ethics Committee; and (3) Cancer S‐183/2015, approved by the Medical Ethics Committee of the University of Heidelberg.

### Participants

Inclusion criteria were histologically confirmed primary OSCC where neck dissection performed as part of primary treatment. All patients were pN‐staged according to the TNM8 classification. Patients with distant metastases (M1) at diagnosis were excluded (n = 2), resulting in a total of 1080 patients. No patients were lost to follow‐up and survival status was obtained through clinical records and reflects the last documented assessment.

### Variables

The primary outcome was overall survival at 3‐ and 5‐year post‐dissection. The main exposure was LNY obtained from the primary surgery and considered in different contexts: treated as a continuous variable, a binary variable using the 18LNY cut‐off (≥18 vs <18), and a categorical variable divided into five yield ranges—elaborated below. We included the following confounders: age on time of neck dissection, sex, neck dissection laterality (ipsilateral or bilateral), number of pN‐positive lymph nodes, status on extranodal extension (ENE), pT‐stage, and application of adjuvant radio‐chemotherapy. Detailed information on the extent of neck dissection (eg, radical, marginal, elective) was unavailable, which is acknowledged as a limitation.

### Bias

Several measures were considered to mitigate bias. We first implemented a cross‐validation strategy by training models on the Heidelberg cohort and evaluating their predictive performance in the independent Cork and Brescia cohorts, mitigating potential bias arising from center‐specific differences. We then employed a counterfactual framework to estimate the average treatment effect using *G*‐computation with inverse probability of censoring weighting (IPCW) to account for informative censoring and confounding arising from different local practices across the three centers.

### Statistical Methods

Time since neck dissection was used as the underlying time scale. Overall survival was defined as the time from neck dissection until death, with patients censored at the time of the last follow‐up if alive.

#### Predictive Performance of LNY

We used all data in this analysis (pooled). The area under the receiver operating characteristic (ROC) curve (AUC) was used to evaluate the accuracy of LNY and 18LNY in predicting the 3‐ and 5‐year mortality.^17^ ROC curves were generated by incorporating LNY as a continuous covariate in subgroups defined from the Union for International Cancer Control (UICC) Stage—here categorized as Stage I‐II and Stage III‐IV—and neck dissection laterality (ipsilateral or bilateral). The 18LNY was identified on the ROC curve.

#### Cross‐Validating the Predictive Performance

The accuracy of LNY and 18LNY in predicting the 3‐ and 5‐year mortality was cross‐validated using the independent cohorts. We applied multiple binomial regression models adjusted to confounders: age, sex, chemoradiotherapy, UICC Stage, ENE, and neck dissection laterality. The model was fitted using inverse probability of censoring weighting, with the censoring weights computed using the Kaplan‐Meier method.^18^

First, three binomial regression models were trained using *only* data from the Heidelberg cohort (as it was the largest). The three models were equivalently adjusted to the covariates described above, but was differentiated by whether they were adjusted to LNY, LNY18, or none of these: (model 1) included LNY as a continuous covariate, (model 2) included the 18LNY, and (model 3) comprised a model without LNY (but otherwise adjusted as described). Secondly, each of the three models were applied to predict the 3‐ and 5‐year risk of death in the Brescia and Cork cohorts, separately; thereby obtaining the AUC measure.

#### Factors Associated With Total Number of LNY

We assessed factors influencing LNY using a multivariable linear regression model with LNY as the outcome, adjusted for each individual center, UICC stage, neck dissection laterality, and an interaction term between UICC stage and neck dissection laterality to better account for more aggressive surgery in more advanced stages of disease.

#### Population‐Average Treatment Effect of LNY

The goal of this analysis was to estimate the population‐average effect of LNY on 3‐year mortality. Using *G*‐computation, we estimated the population‐average treatment effect of LNY ‐ that is, the average risk of death under hypothetical scenarios in which patients were assigned to specific LNY ranges, averaging over the distribution of covariates in the study population. By accounting for confounding factors, this method estimates the effect of obtaining different ranges of LNYs, resembling what *would have* been observed in a randomized trial where OSCC patients were randomly assigned to have a LNY within a specific range independently of their individual patient characteristics. As such, the resulting estimates represent population‐average effects rather than individual‐level associations.^19^ For this analysis, LNY ranges were categorized into five approximately equal groups for pragmatic reasons (each containing between 214 and 223 patients): *LNY 2‐17*, *LNY 17‐25*, *LNY 25‐33*, *LNY 33‐44*, and *LNY 44‐119*. The *LNY 33‐44* was chosen as reference, so both lower and higher LNY groups could be compared. The 3‐year risk of death was selected as the outcome (so the median follow‐up time was met across the individual studies; Table 1). We used a multivariable binomial regression model, which was fitted via inverse probability of censoring weighting, and adjusted to: age, sex, LNY, ENE, UICC stage (I + II and III + IV), neck dissection laterality, whether chemoradiotherapy was received, and each study. To better account for center‐specific practices, we also included a series of interaction terms: (1) each center and UICC Stage, (2) each center and neck dissection laterality, (3) each center and LNY, and (4) each center chemoradiotherapy. Further, two additional interaction terms were included to address treatment heterogeneity: (5) an interaction between UICC stage and neck dissection laterality to account for more extensive surgeries being more common in advanced disease stages, as bilateral neck dissections are typically performed in more aggressive or midline‐involving tumors, and finally (6) we included an interaction between neck dissection laterality and LNY to account for the potential differential impact of LNY depending on whether the dissection was unilateral or bilateral.

**Table 1:** Detailed overview of characteristics of the individual studies

	Test cohort	Validation cohorts		
	Heidelberg	Cork	Brescia	Total
	n = 581	n = 261	n = 238	n = 1080
Person‐years (cohort total),	1888 p‐yrs	1147 p‐yrs	965 p‐yrs	4000 p‐yrs
Median FU years (95% CI)^a^	3.6 (3.3 to 4.0)	6.8 (5.7 to 8.1)	5.4 (4.8 to 6.4)	4.6 (4.3 to 4.9)
LNY/pN‐positive LN	LNY: 25 (2 to 92)	LNY: 30 (3 to 116)	LNY: 43 (7 to 119)	Sum LNY: 35,248
Median (range)	pN^+^: 0 (0 to 21)	pN^+^: 0 (0 to 19)	pN^+^: 0 (0 to 20)	Sum pN^+^: 1423
№ death (cohort total)	127	137	90	354 of 1080 (32.8%)
Death at: 3/5 years post‐surgery	103/112	84/106	62/74	249/292
pT‐stage				
pT1	203	96	45	344 (31.9%)
pT2	159	87	69	315 (29.2%)
pT3	49	18	8	75 (6.9%)
pT4a	87	60	116	263 (24.4%)
pT4b	83	0	0	83 (7.7%)
pN‐stage				
pN0	389	145	125	659 (61.0%)
pN1	59	40	30	129 (11.9%)
pN2a	5	9	8	22 (2.0%)
pN2b	56	18	21	95 (8.8%)
pN2c	32	3	4	38 (3.6%)
pN3a	0	0	0	0 (0.0%)
pN3b	41	46	50	137 (12.7%)
18LNY				
LNY < 18	164	53	6	223 (20.6%)
LNY ≥ 18	417	208	232	857 (79.4%)

^a^ Estimated using the “reverse” Kaplan‐Meier method, which is needed to accurately estimate follow‐up duration in time‐to‐event studies, as simply taking the median follow‐up time with the event as the endpoint underestimates duration by ignoring censored patients who have not experienced the event.^20^, ^21^

The censoring weights were computed using the Kaplan‐Meier method stratified on each individual study.

### Sensitivity Analysis on pT‐Stage in the Prediction Models

In this study, *all* patients were pN‐staged according to the TNM8 classification. For pT‐staging, 62.5% of the OSCC patients (n = 675) were classified according to TNM8; however, the remaining 37.5% were classified using TNM7. In total, 446 patients (41.3%) had pT‐staging information available for both TNM7 and TNM8, thereby enabling a sensitivity analysis to assess the feasibility of merging pT7 and pT8 for simplicity in this study focusing on LNY. The analysis showed that including pT‐stage improved the accuracy of predicting death, but there was no significant difference in predictive accuracy between TNM7 and TNM8. This finding justified merging pT7 and pT8 for practical purposes in this study (referred to as pT‐stage going forward, and includes pT‐stage I, II, III and IV).

## Results

### Descriptive and Outcome Data

The entire cohort of 1080 OSCC patients was followed for 4000 person‐years, during which 354 (32.8%) OSCC patients died, and rendered a median follow‐up of 4.6 years.^20^, ^21^ In total, 35,248 lymph nodes were resected with 1423 categorized as pN‐positive. A total of 421 patients (39.0%) had at least one pN‐positive lymph node. Table 1 provides detailed information pT‐ and pN‐stage, 18LNY categories and outcome data summarized as well as for each individual cohort. Table 2 shows baseline tumor and treatment characteristics stratified by pN‐status and across the three participating centers.

**Table 2:** Baseline clinical characteristics stratified by pN status across participating centers

	Brescia (n = 238)		Cork (n = 261)		Heidelberg (n = 581)	
	pN‐negative	pN‐positive	pN‐negative	pN‐positive	pN‐negative	pN‐positive
Characteristics	n = 125	n = 113	n = 145	n = 116	n = 389	n = 192
Age (median, IQR*)	65 (55‐75)	63 (55‐74)	61 (52‐68)	64 (56‐71)	64 (56‐73)	66 (57‐73)
T‐site						
Alveolar ridge	12 (10%)	12 (11%)	19 (13%)	11 (9%)	100 (26%)	64 (33%)
Buccal mucosa	12 (10%)	5 (4%)	8 (6%)	12 (10%)	37 (10%)	14 (7%)
Floor of mouth	29 (23%)	22 (19%)	44 (30%)	33 (28%)	94 (24%)	42 (22%)
Hard palate	2 (2%)	0 (0%)	2 (1%)	0 (0%)	40 (10%)	15 (8%)
Lip	1 (1%)	0 (0%)	0 (0%)	3 (3%)	0 (0%)	0 (0%)
Retromolar Trigone	13 (10%)	12 (11%)	11 (8%)	10 (9%)	0 (0%)	0 (0%)
Tongue	56 (45%)	62 (55%)	61 (42%)	47 (41%)	95 (24%)	46 (24%)
Soft palate (anterior)	0 (0%)	0 (0%)	0 (0%)	0 (0%)	23 (6%)	11 (6%)
UICC stage						
Stage I and II	75 (60%)	0 (0%)	115 (79%)	0 (0%)	274 (70%)	0 (0%)
Stage III and IV	50 (40%)	113 (100%)	30 (21%)	116 (100%)	115 (30%)	192 (100%)
Radiochemotherapy						
Received	53 (42%)	95 (84%)	64 (44%)	82 (71%)	76 (20%)	144 (75%)
Not received	72 (58%)	18 (16%)	81 (56%)	34 (29%)	313 (80%)	48 (25%)

Abbreviation: IQR, the 1st and 3rd quartile.

### Main Results

#### Predictive Performance of LNY

**Figure 1:**
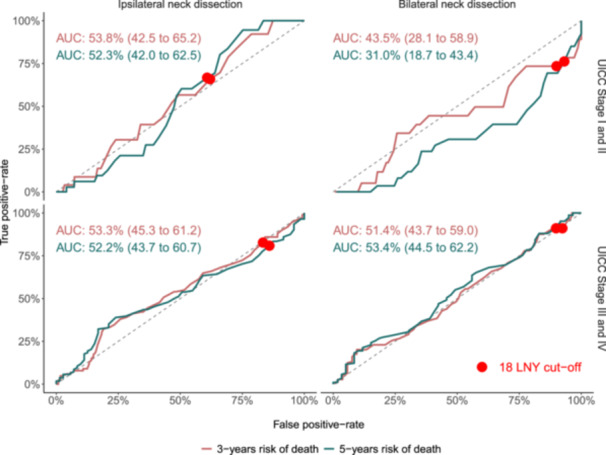
ROC curves and AUC values showing prognostic value of adding 18LNY to UICC stage and neck dissection laterality in predicting 3‐ and 5‐year mortality. 18LNY is highlighted across all strata.

We used a stratification of UICC Stage and neck dissection laterality and used to predict the 3‐ and 5‐years risk of death (Figure 1). LNY was added to each stratum and, based on the corresponding AUC percentages from the ROC curve, LNY did not improve the prognostic accuracy beyond what was already captured within the specific stratum.

Incorporating LNY into the stratum of UICC Stage I‐II lesions with bilateral neck dissection suggested a poor predictive performance. This outcome may be expected, as bilateral neck dissection in lower‐grade lesions might only apply in certain clinical cases, such as midline or near‐midline tumors; bilateral neck dissection may not reflect more advanced disease stages in this subgroup.

#### Cross‐Validating the Predictive Performance

The accuracy of the three adjusted binomial regression models trained on the Heidelberg cohort was evaluated to predict 3‐ and 5‐year mortality in the Brescia and Cork cohorts, separately.

**Figure 2:**
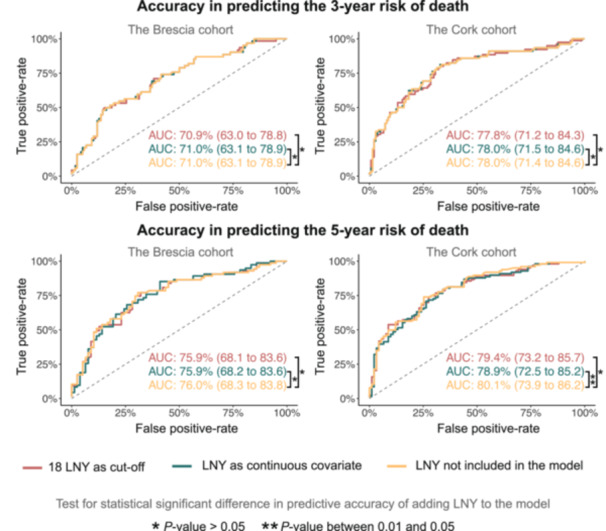
Binomial models trained in Heidelberg and tested in Brescia and Cork cohorts. Compared models include (1) no LNY, (2) continuous LNY, and (3) 18LNY. All models were adjusted identically for remaining covariates.

The ROC curves and corresponding AUC percentages indicated that adding either LNY or 18LNY did not improve predictive accuracy once the included covariates had been adjusted to in the “LNY‐naïve” model (Figure 2). This finding was consistent across both cohorts and for the 3‐ and 5‐year mortality predictions.

#### Factors Associated With Total Number of LNY

Table 3 compares mean LNY across cohorts, demographic factors, and neck dissection laterality. The Brescia and Cork cohorts show significantly higher LNY compared to the Heidelberg cohort, with Brescia at 23.6 (95% CI: 21.2 to 26.1, *P* < 0.0001) and Cork at 13.1 (95% CI: 10.7 to 15.4, *P* < 0.0001). Supplemental Figure S1, available online shows the LNY within TNM8 pN‐groups stratified for each center.

**Table 3:** Multivariable linear regression used to estimate the mean LNY with 95% confidence intervals (95% CI) and *P*‐values. In addition to each study cohort, the model was adjusted for UICC stage, neck dissection laterality, study cohort, age (per 10‐year increase), and sex. We included an interaction term between neck dissection laterality and UICC stage to account for extensive surgery in more advanced stages of disease

Covariate	Mean LNY (95% CI, *P*‐value)
Heidelberg cohort	Reference
Brescia cohort	23.6 (21.2 to 26.1, *P* < 0.0001)
Cork cohort	13.1 (10.7 to 15.4, *P* < 0.0001)
Age at surgery per 10‐year increase	−0.9 (−1.7 to −0.2, *P* = 0.01)
Female	Reference
Male	4.0 (2.2 to 5.8, *P* < 0.0001)
Neck dissection laterality Ipsilateral	
II vs I	2.8 (−1.0 to 6.6, *P* = 0.2)
III vs I	1.9 (−2.5 to 6.3, *P* = 0.4)
IVa vs I	6.8 (3.3 to 10.2, *P* < 0.001)
IVb vs I	9.3 (5.3 to 13.3, *P* < 0.0001)
Bilateral	
II vs I	−0.0 (−4.0 to 4.0, *P* = 1.0)
III vs I	−0.3 (−5.1 to 4.4, *P* = 0.9)
IVa vs I	4.0 (0.5 to 7.5, *P* = 0.03)
IVb vs I	6.0 (2.5 to 9.6, *P* < 0.001)

Age at surgery is associated with a decrease in LNY per 10‐year increase in age at −0.9 (95% CI: −1.7 to −0.2, *P* = 0.01), while males have significantly higher LNY than females at 4.0 (95% CI: 2.2 to 5.8, *P* < 0.0001). For neck dissection laterality, both ipsilateral neck dissection in UICC stages IVa and IVb (6.8 [95% CI: 3.3 to 10.2, *P* < 0.001]; and, 9.3 [95% CI: 5.3 to 13.3, *P* < 0.0001]) and bilateral neck dissection in UICC stages IVa and IVb (4.0 [95% CI: 0.5 to 7.5, *P* = 0.03]; and, 6.0 [95% CI: 2.5 to 9.6, *P* < 0.001]) show significant increases in LNY compared to stage I, indicating that advanced stages are associated with a higher LNY, regardless type of dissection (Table 3).

#### Population‐Average Treatment Effect of LNY

The risk of death was compared across patients *who would* have randomly received LNY within that specific range (LNY: *2‐17*, *17‐25*, *25‐33*, *33‐44*, and *44‐119*), corresponding to approximately equal groups in size (between 214 and 223 patients).

**Figure 3:**
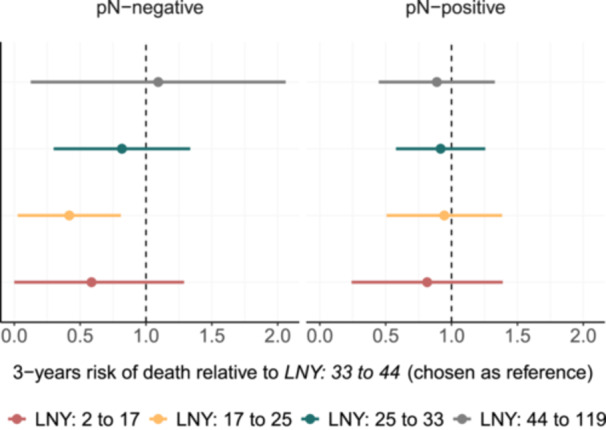
The estimated 3‐year mortality risk across LNY categories using a population‐average treatment effect model, adjusted for key covariates including interaction terms to account for cohort‐specific effects. Reference: LNY 33–44, indicated by dashed line.

The average treatment effect model was applied to pN‐negative and pN‐positive patients, separately (Figure 3). Using the *LNY 33‐44* group as the reference, the 3‐year risk of death was not significantly different across any LNY category for pN‐positive patients when compared to the reference group (Figure 3). For pN‐negative patients, the 3‐year risk of death was significantly lower in the LNY 2‐17 groups compared to the reference group (ratio of predicted risks: 0.42, 95% CI: 0.03 to 0.80, *P* = 0.004). This suggests that a lower LNY was associated with better survival probability in this subgroup compared to the reference group *LNY 33‐44*; however, the estimate had a considerably wide 95% confidence interval. Moreover, no significant differences were observed for the remaining LNY groups, indicating no additional survival benefit from higher LNYs beyond the reference range (Figure 3).

### Other Analysis

#### Exploratory Post Hoc Analysis: Assessment of Potential Publication Bias

**Figure 4:**
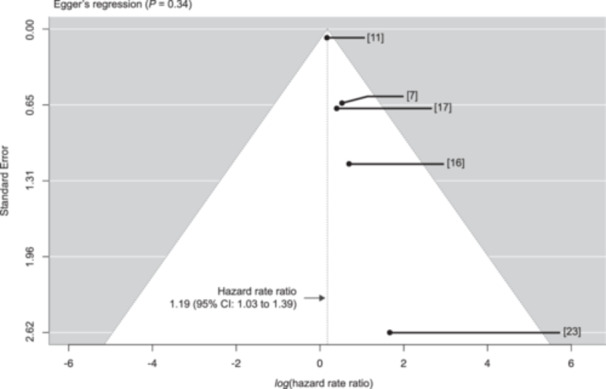
Effect estimates from five studies assessing LNY cut‐offs (≥18–21) and prognosis in head and neck cancer. A random‐effects model estimated mean hazard ratios; four studies focused on OSCC.

Since LNY did not improve predictive accuracy in this present study, we conducted an exploratory post hoc analysis to assess potential publication bias. This focused on studies that reported hazard rate ratios from multivariable Cox regression models, specifically including those that used a high LNY cut‐off as the reference group (eg, ≥18 (reference) vs <18). To evaluate the consistency and reliability of these effect estimates, we constructed a funnel plot based on log hazard rate ratios and their standard errors, and fitted a random‐effects model using restricted maximum likelihood estimation (Figure 4).

Five studies were included in this analysis: four focused specifically on OSCC, and one addressed head and neck cancer more broadly.^7^, ^10^, ^15^, ^16^, ^22^ The LNY cut‐offs ranged from 18 to 21, and while most adjusted for pN‐status, one study applied multivariable Cox regression only to pN‐positive patients. While visual inspection of the funnel plot suggested possible asymmetry toward studies reporting a survival benefit when the LNY cut‐off was achieved, Egger's regression test did not provide statistical evidence of funnel plot asymmetry (*P* = 0.34). However, this formal test may have limited sensitivity in this setting due to the inclusion of one very large study (n = 63,978 ^10^) alongside several smaller ones (n = 78 to 1567^7^, ^15^, ^16^, ^22^), which may influence the regression estimate and obscure subtle asymmetry patterns (ie, heteroscedasticity arising from markedly different standard errors due to considerable differences in cohort sizes).

Additional studies using the *lower* LNY cut‐off as the reference group (eg, <18 (reference) vs ≥18) were not eligible for inclusion in the funnel plot analysis, but similarly reported positive effects associated with achieving a higher LNY. Across these studies, a survival benefit of higher LNYs is suggested in various contexts, including OSCC, HPV‐associated oropharyngeal cancer, and laryngectomized patients without cervical metastasis.^4^, ^11^, ^23^, ^24^

While consistent reporting of favorable outcomes might reflect a true effect, it could also indicate selective publication. To further explore this, we reviewed studies with null findings. One head and neck cancer study reported an insignificant hazard rate ratio of 1.10 (95% CI: 0.87–1.40) for disease‐specific survival when comparing <19 LNY vs ≥34 LNY.^25^ Another OSCC study found an insignificant hazard rate ratio of 1.14 (95% CI: 0.16‐8.29) for <18 vs ≥18 LNY in univariate analysis.^26^ In both cases, the authors excluded the LNY cut‐off from their final multivariable Cox models due to lack of significance in univariate analysis.^25^, ^26^

Taken together, although Egger's regression did not indicate statistical asymmetry, the visual skew in the funnel plot, consistent positive findings across studies, and potential reporting exclusions suggest that some degree of publication bias cannot be ruled out nor can it be confirmed.

## Discussion

### Key Findings

Indicated by AUC percentages, we did not observe that LNY (including 18LNY) provided any prognostic information on top of what was captured when stratifying patients according to UICC Stage and neck dissection laterality. Similarly, LNY did not improve predictive accuracy when added to predict the 5‐year probability of death using a binomial regression model adjusted to age, sex, UICC stage, neck dissection laterality, ENE, and application of chemoradiotherapy.

Furthermore, we estimated the population‐average treatment effect of LNY using different LNY categories, which were obtained by dividing patients into approximately equally sized groups. The goal was to estimate the population‐average effect of LNY on overall survival by simulating what would have been observed if OSCC patients had been randomly assigned to receive neck dissections yielding lymph nodes within specific predefined ranges, irrespective of their individual characteristics. This strategy was applied to both pN‐negative and pN‐positive patients, with no evidence in either group supporting LNY as prognostically favorable.

### Interpretation

LNY is a straightforward and widely used metric across many surgical specialties, but its interpretation is rather complex due to variability related to factors that are difficult to fully account for. This variability may arise from differences in clinical practices, population characteristics, diagnostic guidelines, and center‐specific treatment protocols—all of which may influence LNY and therefore complicate its use as a standardized prognostic tool.^27^ In this study, despite adjusting for UICC stage, neck dissection laterality, age, and sex in a multivariable linear regression model, the mean LNY still differed significantly across the Brescia, Cork, and Heidelberg cohorts. This indicates that factors beyond these common confounders influenced the total number LNY in this study, suggesting that LNY is highly context‐specific and may ultimately complicate direct comparisons across studies. While common confounders like tumor stage can be adjusted for in analysis, cohort‐specific factors, including differences in surgical techniques, experience and the expertise of surgeons, pathologists, and radiotherapists, can be difficult to fully account for. Furthermore, patient‐specific information may further affect LNY, such as body mass index, but is rarely recorded and included for survival analysis utilizing LNY.^1^

Our results demonstrate that LNY does not improve individual‐level prognostication. This suggests that using LNY—including arbitrary thresholds such 18LNY—may have limited utility in accurately identifying high‐risk patients. Beyond methodological concerns, applying a universal LNY threshold to define high‐risk patients also introduces practical challenges. Any binary cut‐off ‐ such as low‐/high‐risk categorization derived from the 18LNY—will inevitably produce false‐positive and false‐negative classifications. As such, the utility of cut‐off values must be weighed not only in terms of prognostic performance but also in light of the clinical consequences of misclassification. An imbalanced rate of false‐positives may lead to unnecessary diagnostic procedures or overly intensified surveillance, whereas false‐negatives may delay essential care. In a real‐world oncological setting, false‐negatives—that is, patients incorrectly deemed low‐risk—carry particularly serious implications and their mitigation will always be prioritized at the cost of higher false‐positive rates. The direct consequence of increasing false‐positives will affect healthcare systems unevenly depending on their structure and capacity. For instance, insurance‐based or resource‐constrained systems may be disproportionately burdened by an influx of patients incorrectly flagged as high‐risk. This underscores the importance of evaluating any proposed cut‐off not isolated in terms of predictive performance, but also in the context of both clinical utility and system‐level feasibility.

A previous investigation into head and neck cancer quality metrics supported the use of a ≥ 18 LNY threshold as a surgical quality indicator (due to its association with improved survival).^24^ However, the study did not consider the downstream impact of false‐positive and false‐negative classifications introduced by rigid cut‐offs, nor do they assess the feasibility of applying such thresholds across heterogeneous healthcare systems. In contrast, our findings emphasize the methodological limitations and potential clinical consequences of using fixed LNY thresholds to define high‐risk patients remain unestablished. Given the lack of added prognostic value, the substantial inter‐institutional variability, and the absence of evidence on healthcare system‐level consequences, we do not find sufficient justification to support LNY cut‐off values as a universal surgical quality metric.

### Comparison With Previous Literature

Cox proportional hazards models and Kaplan‐Meier survival analysis are commonly used methods for determining prognostic value of LNY cut‐offs.^7^, ^8^, ^10^, ^14^, ^15^, ^16^, ^22^, ^28^, ^29^ When a Kaplan‐Meier analysis is used to derive a LNY cut‐off, the resulting threshold can be somewhat arbitrary, simply just reflecting the specific demographics and characteristics of the study population used. A lower LNY may appear to correlate with poor survival, not due to a direct effect of LNY on survival probability, but because of confounding factors that are not accounted for. Even when Kaplan‐Meier‐derived cut‐offs are applied to an adjusted Cox model to control for these confounders, the resulting cut‐off may still be shaped by cohort‐specific characteristics, potentially limiting its broader applicability. Both methods are powerful for identifying associations between LNY groups and survival outcomes, however, neither of them is inherently designed to determine or evaluate a cut‐off value. Statistical techniques such as ROC analysis and measures of predictive accuracy, such as AUC, are more appropriate but infrequently used.^7^, ^22^

Ebrahimi et al. were the first to propose the 18‐lymph node cut‐off in their 2011 study, which has since gained widespread use.^15^ The number 18 emerged from categorizing LNY based on quartiles, which were: <18, 18 and <24, 24 and <32, and ≥32. Then, in a Kaplan‐Meier plot, the authors observed that patients with <18 LNY had descriptively poorer survival probabilities. This descriptive pattern was further supported by an unadjusted Cox regression model, prompting the authors to collapse the grouping into a binary classification of <18 vs ≥ 18 LNY. However, the selection of 18 was essentially data‐driven and contingent on how the initial quartiles split, making it completely arbitrary. As a result, while the 18LNY threshold has become influential, it originated from within‐cohort descriptive trends rather than rigorous methodological evaluation.

While previous literature has demonstrated associations between higher LNY and improved outcomes, our findings suggest that these associations may reflect context‐specific characteristics or uncontrolled confounding rather than true prognostic value.^7^, ^8^, ^10^, ^14^, ^15^, ^16^, ^28^, ^29^

We recognize that LNY may correlate with survival in certain data sets; however, our analysis suggests that such associations do not necessarily translate into generalizable or robust prognostic performance across cohorts. By incorporating external validation and population‐average effect estimation, our approach seeks to evaluate the prognostic value of LNY in a way that may better reflect a real‐world clinical setting. While LNY may, in some settings, act as a surrogate for surgical thoroughness or pathology diligence, its broader application as a universal prognostic biomarker or quality metric appears limited.

These findings challenge the assumption that LNY holds consistent prognostic meaning across centers and support a more critical, context‐aware interpretation of earlier studies. We believe this study contributes to refining how LNY thresholds should be viewed in clinical and benchmarking practices, particularly when used to define surgical adequacy or guide treatment intensity.

### Strengths and Limitation

The major strength of this present study was the ability to train and cross‐validate prognostic models in independent OSCC cohorts. Further, this enabled investigation of how LNY was correlated to parameters such as different cohorts, age and TNM Stage. The availability of independent OSCC cohorts ensured generalizability rather than estimating effect from single cohorts.

There were limitations in the presented study. Information on type of neck dissection (eg, radical, marginal, elective) was unavailable, which is acknowledged as a limitation. However, we applied an interaction term between UICC stage and neck dissection laterality (ipsilateral or bilateral) in the analysis of LNY and death. Advanced disease stages (UICC Stage III‐IV) often require more extensive surgery, which tends to involve bilateral neck dissections more frequently. By modeling this interaction, potential biases are likely reduced by accounting for the combined effects of disease severity and surgical approach, ensuring that the model reflects the clinical decision‐making that correlates with both cancer stage and surgical extent. While we adjusted for center‐specific practices through interaction terms, we did not have information on the individual surgeons who performed the neck dissections. As LNY may vary by surgeon due to differences in technique, experience, or training, this is a potential source of variation that we were unable to address directly. Although center‐level adjustment captures some of the institutional differences, variation at the level of individual providers could still contribute to residual confounding.

Furthermore, differences in pathological assessment and reporting practices across institutions may have contributed to variability in the recorded LNY. In the absence of a centralized pathology review or standardized nodal reporting protocol across sites, we cannot fully rule out the influence of inter‐institutional differences in specimen handling, embedding, and node counting procedures. Likewise, data collection was performed retrospectively at each site without a predefined framework or uniform data abstraction sheet. Although the final data set was harmonized for analysis, institutional differences in documentation and interpretation may have introduced additional inconsistencies. These limitations reflect real‐world heterogeneity and underscore the challenges in using LNY as a reproducible metric across clinical settings and multi‐institutional studies.

While the vast majority of patients was pT‐staged according to TNM8, around 37.5% were pT‐staged using TNM7. A sensitivity analysis, however, found no prognostic differences between pT7 and pT8. Though it would have been ideal to stage all patients under TNM8, we believe that reclassification would not have affected the results.

In comparison to existing literature, previous studies on LNY cut‐off values have included patients since the 1970s, implying that diagnostics of these patients span more than four decades, during which different staging algorithms were used. In this context, the discussed limitations are considered negligible.^16^

## Conclusions

Utilizing data from three independent OSCC cohorts, we did not observe any improvement in the predictive accuracy for death when considering LNY or 18LNY. While a higher LNY has previously been associated with improved survival outcomes, our findings do not support its use as a reliable prognostic marker when adjusting for established clinical variables. Moreover, the substantial inter‐cohort variability and lack of generalizable prognostic value challenge the clinical utility of fixed LNY cut‐offs such as the 18LNY. These results highlight the need for a context‐aware interpretation of LNY in clinical decision‐making and when considering its use as a surgical quality metric.

## Author Contributions

**Christian Mirian** and **Therese Ovesen**, led the collaboration, including the analysis; **Davide Mattavelli**, **Cesare Piazza**, **Linda Feeley**, **Patrick Sheahan**, **Marcel Oehme**, **Christian Freudlsperger**, and **Julius Moratin**, collected data. All authors contributed to interpreting results and writing the manuscript. All authors have approved the current edition.

## Disclosures

### Competing interests

The authors have nothing to disclose.

### Funding source

No funding was received for this project.

## Supporting information

SupplementaryFigure1_LNY.

## References

[1] Hanberg P , Tramm T , Pikelis A , Schytte S , Gade SD , Klug TE . Factors affecting lymph node yield and density in neck dissection. Acta Otolaryngol. 2024;144:379‐383. 10.1080/00016489.2024.2380863 39041248

[2] Huang L , Huang T , Li L , et al. Factors associated with lymph node yield and effects of lymph node density on survival of patients with pulmonary sarcomatoid carcinoma. Am J Clin Oncol. 2022;45(11):458‐464. 10.1097/COC.0000000000000946 36256867 PMC9624378

[3] Pou JD , Barton BM , Lawlor CM , Frederick CH , Moore BA , Hasney CP . Minimum lymph node yield in elective level I–III neck dissection. Laryngoscope. 2017;127(9):2070‐2073. 10.1002/LARY.26545 28271566

[4] Zenga J , Stadler M , Massey B , et al. Lymph node yield from neck dissection in HPV‐associated oropharyngeal cancer. Laryngoscope. 2020;130(3):666‐671. 10.1002/LARY.28102 31206708

[5] Gomez ED , Chang JC , Ceremsak JJ , et al. Impact of lymph node yield on survival in surgically treated oropharyngeal squamous cell carcinoma. Otolaryngol Head Neck Surg. 2020;164(1):146‐156. 10.1177/0194599820936637 32689888

[6] Feng Z , Qin LZ , Huang X , Li JZ , Su M , Han Z . Nodal yield: Is it a prognostic factor for head and neck squamous cell Carcinoma? J Oral Maxillofac Surg. 2015;73(9):1851‐1859. 10.1016/j.joms.2015.03.027 25871901

[7] Stampe H , Jakobsen KK , Tvedskov JF , et al. Prognostic Value of Lymph Node Yield, Lymph Node Density, and pN in Oral Cancer. Otolaryngol Head Neck Surg. 2022;169(2):276‐285. 10.1177/01945998221123927 36066971

[8] Voss JO , Freund L , Neumann F , et al. Prognostic value of lymph node involvement in oral squamous cell carcinoma. Clin Oral Investig. 2022;26(11):6711‐6720. 10.1007/S00784-022-04630-7/FIGURES/3 PMC9643253 35895143

[9] Divi V , Harris J , Harari PM , et al. Establishing quality indicators for neck dissection: Correlating the number of lymph nodes with oncologic outcomes (NRG Oncology RTOG 9501 and RTOG 0234. Cancer. 2016;122(22):3464‐3471. 10.1002/CNCR.30204 27419843 PMC5237619

[10] Divi V , Chen MM , Nussenbaum B , et al. Lymph node count from neck dissection predicts mortality in head and neck cancer. J Clin Oncol. 2016;34(32):3892‐3897. 10.1200/JCO.2016.67.3863 27480149

[11] Böttcher A , Dommerich S , Sander S , et al. Nodal yield of neck dissections and influence on outcome in laryngectomized patients. Eur Arch Otorhinolaryngol. 2016;273(10):3321‐3329. 10.1007/S00405-016-3928-2 26874731

[12] Kuo P , Mehra S , Sosa JA , et al. Proposing prognostic thresholds for lymph node yield in clinically lymph node‐negative and lymph node‐positive cancers of the oral cavity. Cancer. 2016;122(23):3624‐3631. 10.1002/CNCR.30227 27479645

[13] Cheraghlou S , Otremba M , Kuo Yu P , Agogo GO , Hersey D , Judson BL . Prognostic Value of Lymph Node Yield and Density in Head and Neck Malignancies. Otolaryngol Head Neck Surg. 2018;158(6):1016‐1023. 10.1177/0194599818756830 29460685

[14] Lemieux A , Kedarisetty S , Raju S , Orosco R , Coffey C . Lymph node yield as a predictor of survival in pathologically node negative oral cavity carcinoma. Otolaryngol Head Neck Surg. 2016;154(3):465‐472. 10.1177/0194599815622409 26701177

[15] Ebrahimi A , Zhang WJ , Gao K , Clark JR . Nodal yield and survival in oral squamous cancer: Defining the standard of care. Cancer. 2011;117(13):2917‐2925. 10.1002/cncr.25834 21246523

[16] Ebrahimi A , Clark JR , Amit M , et al. Minimum nodal yield in oral squamous cell carcinoma: defining the standard of care in a multicenter international pooled validation study. Ann Surg Oncol. 2014;21(9):3049‐3055. 10.1245/S10434-014-3702-X 24728823

[17] Gerds TA , Cai T , Schumacher M . The Performance of Risk Prediction Models. Biometrical J. 2008;50(4):457‐479. 10.1002/bimj.200810443 18663757

[18] Blanche PF , Holt A , Scheike T . On logistic regression with right censored data, with or without competing risks, and its use for estimating treatment effects. Lifetime Data Anal. 2023;29(2):441‐482. 10.1007/S10985-022-09564-6/FIGURES/3 35799026

[19] Hernán M , Robins J . Causal Inference: What If. Chapman & Hall/CRC; 2020.

[20] Moore DF . Applied survival analysis using R. Published online 2016. 10.1007/978-3-319-31245-3

[21] Schemper M , Smith TL . A note on quantifying follow‐up in studies of failure time. Control Clin Trials. 1996;17(4):343‐346. 10.1016/0197-2456(96)00075-X 8889347

[22] Lee S , Kim HJ , Cha IH , Nam W . Prognostic value of lymph node count from selective neck dissection in oral squamous cell carcinoma. Int J Oral Maxillofac Surg. 2018;47(8):953‐958. 10.1016/J.IJOM.2018.03.007 29606561

[23] Gomez ED , Chang JC , Ceremsak JJ , et al. Impact of lymph node yield on survival in surgically treated oropharyngeal squamous cell carcinoma. Otolaryngol Head Neck Surg. 2021;164(1):146‐156. 10.1177/0194599820936637 32689888

[24] Cramer JD , Speedy SE , Ferris RL , Rademaker AW , Patel UA , Samant S . National evaluation of multidisciplinary quality metrics for head and neck cancer. Cancer. 2017;123(22):4372‐4381. 10.1002/CNCR.30902 28727137

[25] Feng Z , Qin LZ , Huang X , Li JZ , Su M , Han Z . Nodal yield: is it a prognostic factor for head and neck squamous cell carcinoma. J Oral Maxillofac Surg. 2015;73(9):1851‐1859. 10.1016/J.JOMS.2015.03.027 25871901

[26] Son HJ , Roh JL , Cho KJ , Choi SH , Nam SY , Kim SY . Nodal factors predictive of recurrence and survival in patients with oral cavity squamous cell carcinoma. Clin Otolaryngol. 2018;43(2):470‐476. 10.1111/COA.12995 28981214

[27] Mirian C , Gerds TA , Pedersen MM , et al. Metrics of pN‐staging in oral squamous cell carcinoma: An analysis of 1,905 patients. Eur J Cancer. 2021;150:33‐41. 10.1016/j.ejca.2021.03.019 33887515

[28] Jaber JJ , Zender CA , Mehta V , et al. Multi‐institutional investigation of the prognostic value of lymph nodel yield in advanced‐stage oral cavity squamous cell carcinoma. Head Neck. 2014;36(10):1446‐1452. 10.1002/HED.23475 24038739 PMC4136977

[29] Kuo P , Mehra S , Sosa JA , et al. Proposing prognostic thresholds for lymph node yield in clinically lymph node‐negative and lymph node‐positive. Cancer. 2016;122(23):3624‐3631. 10.1002/CNCR.30227 27479645

