# Supplementary material for: Lymph Nodal Yield and Prediction of Mortality in Oral Squamous Cell Carcinoma Patients
Source: Otolaryngol Head Neck Surg. 2025 Oct 17;173(6):1421–31. doi: 10.1002/ohn.70040 (PMC12661473; doi:10.1002/ohn.70040)

**Supplementary Figure 1**. The lymph nodal yield from all OSCC patients while stratified for pN-stage and ENT center.


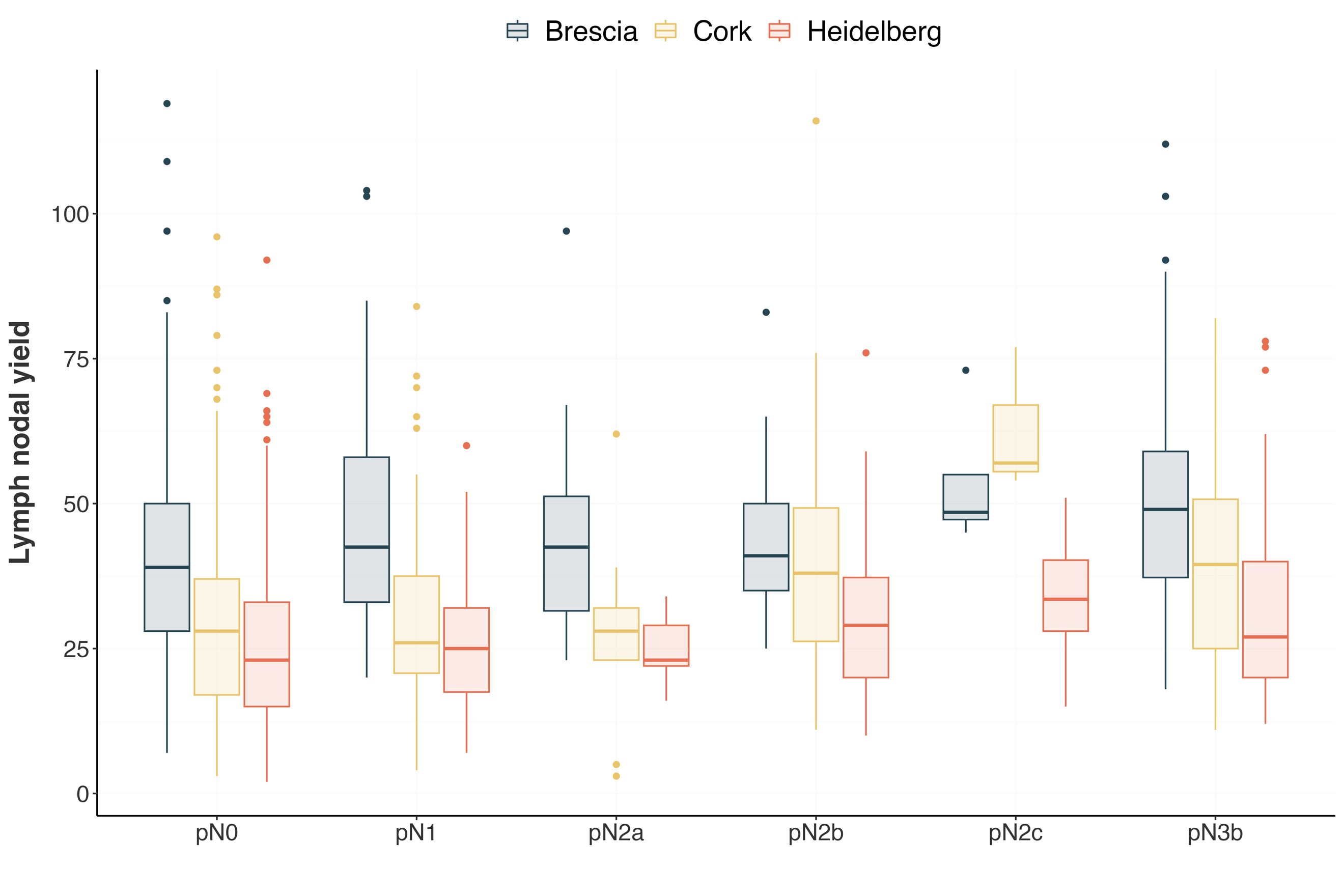

Supplement: Supplementary file 1 — SupplementaryFigure1_LNY. [file OHN-173-1421-s001.docx]
